# 

**Published:** 1864-11

**Authors:** 


### BOOKS AND PAMPHLETS RECEIVED.

*A System of Surgery; Pathological, Diagnostic, Therapeutic and Operative, by* Samuel D. Gross, M. D., *Professor of Surgery in the Jefferson College of Philadelphia; Surgeon to the Philadelphia Hospital; Member of the Imperial Royal Medical Society of Vienna, etc., etc., illustrated by over thirteen hundred engravings. Third edition, much enlarged and carefully revised. In two volumes. Philadelphia:* Blanchard & Lea, 1864.

*Therapeutics and Materia Medica; a Systematic Treatise on the Action, and Uses of Medical Agents, including their Description and History, by* Alfred Stille, M. D., *Professor of the Theory and Practice of Medicine in the University of Pennsylvania; Physician to St. Joseph's Hospital; Fellow of the College of Physicians, and Member of the Pathological Society of Philadelphia; Member of the Societe Medicale D'observation, of Paris; Honorary Member of the Medical Society of the State of Rhode Island, of the State of New York, etc. Second edition, revised and enlarged. In two volumes. Philadelphia:* Blanchard & Lea, 1864.

*Outlines of Surgical Diagnosis, by* George H. B. MacLeod, M. D., F. R. C. S. E.; *El. Fac. Phys. and Surg. Glasgow; Lecturer on Surgery Anderson's University; Surgeon to the Glasgow Boyal Infirmary, and the Lock Hospital; late Senior Surgeon Civil Hospital Smyrna, and General Hospital in Camp before Sebastopol; Mem. Cor. De La Soc. de Chir. de Paris; and author of "Notes on the Surgery of the War in the Crimea." First American edition, reprinted from advance sheets. New York:* Bailliere Brothers, 520 *Broadway,* 1864.

*Annals of the Medical Society of the County of Albany,* 1806—1851, *with Biographical Sketches of Deceased Members, by* Sylvester D. Willard, M. D. *Albany:* J. Munsell, 78 *State Street,* 1864.

*Diphtheria; its Nature and Treatment, with an account of the History of its Prevalence in various countries, by* Daniel D. Slade, M. D., *being a second and revised edition of an Essay to which was awarded the Fiske Fund Prize* 1860. *Philadelphia:* Blanchard & Lea, 1864.

*The Army Surgeon' Manual, for the use of Medical Officers, Cadets, Chaplains, and Hospital Stewards, containing the Regulations of the Medical Department, all General Orders from the War Department, and Circulars from the Surgeon-General's Office, from January* 1*st*, 1861 *to July* 1*st*, 1864, *by* WILLIAM GRACE, *of Washington, D. C. Published by permission of the Surgeon-General. New York:* BAILLIERE BROTHERS, 520 *Broadway*, 1864.

*Transactions of the Medical Society of the State of Pennsylvania, at its Fifteenth Annual Session, held in Philadelphia, June*, 1864. *Third Series—Part* 3. *Published by the Society.*

*A Midsummer's Ride on the Great Lakes, by* WM. WIRT SIKES,

*Address Introductory to the Twenty-Second Annual Course of Lectures in Rush Medical College, by* DE LASKIE MILLER, M. D., *Professor of Obstetrics and Diseases of Women and Children. Published by the Class.*

*Glaucoma; its Symptoms, Diagnosis, and Treatment, by* PETER DIRCK KEYSER, M. D. *Philadelphia:* LINDSAY & BLAKISTON, 1864.

*Report of a Successful Operation in a case of Subclavian Aneurism, by* A. W. SMYTH, M. D., *House Surgeon, Charity Hospital, New Orleans, Louisiana.*

*A New Method for Treating Fractures of the Femur in Children, by* G. D. BEEBE, M. D., *late Surgeon-in-Chief of the* 14*th Army Corps, U. S. A.*

*The Atlantic Monthly, Devoted to Literature, Art and Politics, November*, 1863. *Boston:* TICKNOR & FIELDS.

*Peterson's Ladies' National Magazine, November. Terms*, $2 *a year, in advance.*

*Godey's Lady's Book, Edited by Mrs.* SARH J. HALE *and* L. A. GODEY. *November*, 1864. *For terms, see advertisement.*

